# Salvage pembrolizumab added to kinase inhibitor therapy for the treatment of anaplastic thyroid carcinoma

**DOI:** 10.1186/s40425-018-0378-y

**Published:** 2018-07-11

**Authors:** Priyanka C. Iyer, Ramona Dadu, Maria Gule-Monroe, Naifa L. Busaidy, Renata Ferrarotto, Mouhammed Amir Habra, Mark Zafereo, Michelle D. Williams, G. Brandon Gunn, Horiana Grosu, Heath D. Skinner, Erich M. Sturgis, Neil Gross, Maria E. Cabanillas

**Affiliations:** 1 Department of Endocrine Neoplasia and Hormonal Disorders, The University of Texas MD Anderson Cancer Center, 1515 Holcombe Blvd, Unit 1461, Houston, TX 77030 USA; 2 Department of Diagnostic Radiology, Division of Diagnostic Imaging, The University of Texas MD Anderson Cancer Center, 1515 Holcombe Blvd, Unit 1482, Houston, TX 77030 USA; 3 Department of Thoracic/Head and Neck Medical Oncology, Division of Cancer Medicine, The University of Texas MD Anderson Cancer Center, 1515 Holcombe Blvd, Unit 0432, Houston, TX 77030 USA; 4 Department of Head and Neck Surgery, Division of Surgery, The University of Texas MD Anderson Cancer Center, 1515 Holcombe Blvd, Unit 1445, Houston, TX 77030 USA; 5 Department of Pathology, Division of Pathology/Lab Medicine, The University of Texas MD Anderson Cancer Center, 1515 Holcombe Blvd, Unit 0085, Houston, TX 77030 USA; 6 Department of Radiation Oncology, Division of Radiation Oncology, Proton Therapy Center, 1515 Holcombe Blvd, Unit 0097, Houston, TX USA; 7 Department of Pulmonary Medicine, Division of Internal Medicine, The University of Texas MD Anderson Cancer Center, 1515 Holcombe Blvd, Unit 1462, Houston, TX 77030 USA

**Keywords:** Anaplastic thyroid cancer, Immunotherapy, Salvage, Thyroid cancer, Lenvatinib, Dabrafenib, Trametinib, Undifferentiated

## Abstract

**Background:**

Anaplastic thyroid carcinoma (ATC) is a rare but deadly form of thyroid cancer. Kinase inhibitors kinase inhibitors have shown clinical efficacy in the management of ATC, however, eventually these tumors acquire resistance to KI and patients succumb to their disease. Salvage therapy in this setting is limited. As ATC tumors diffusely express the programmed cell death protein ligand (PD-L1), anti- programmed cell death protein (PD-1) drugs such as pembrolizumab offer therapeutic potential. We sought to explore the efficacy of adding pembrolizumab to kinase inhibitors at progression in ATC.

**Methods:**

We retrospectively reviewed the charts of ATC patients initiated on pembrolizumab in combination with KI at the time of progression on kinase inhibitors at MD Anderson Cancer Center between August 2016 and August 2017. Efficacy was evaluated with best overall response (BOR) using RECISTv1.1 criteria. Progression free survival (PFS) from the start of pembrolizumab and overall survival (OS) from the start of kinase inhibitors, as well as from the time of addition of pembrolizumab were calculated.

**Results:**

Twelve patients were treated with combination kinase inhibitors plus pembrolizumab at the time of progression on their KI therapy. Median age at initiation of pembrolizumab was 60 years (range 47–84 years). BOR was as follows: 5/12 (42%) had partial response, 4/12 (33%) had stable disease and 3/12 (25%) had progressive disease. Median OS from the start of kinase inhibitor was 10.43 months (95% CI = 6.02, 14.83, range 5.4–40 months). Median OS and PFS from the addition of pembrolizumab were 6.93 months (95% CI = 1.7, 12.15, range 3–15.9 months) and 2.96 months (95% CI = 2.2, 3.7, range 0.57–13.14 months), respectively. Fatigue, anemia and hypertension were the most common AEs encountered on these combinations. Therapy had to be discontinued in 2 patients due to drug induced rash and altered mental status likely from progression of disease.

**Conclusion:**

In a subset of ATC patients, pembrolizumab may be an effective salvage therapy added to kinase inhibitors at the time of progression on these drugs. However, better treatment strategies aimed at incorporating immunotherapy in patients with ATC should be explored. Frontline combination of KI with immunotherapy should be studied in prospective clinical trials.

**Electronic supplementary material:**

The online version of this article (10.1186/s40425-018-0378-y) contains supplementary material, which is available to authorized users.

## Background

Anaplastic thyroid carcinoma (ATC) is rare, but the most aggressive form of thyroid cancer, accounting for fewer than 2% of all thyroid cancers but responsible for more than a third of deaths due to thyroid cancer [1]. Median overall survival in these patients has reportedly been around 5 months with a 1 year mortality of 80% [2]. Clinically, these patients present with very rapidly growing, large tumors, often causing compressive symptoms such as dysphagia and stridor.

Current guidelines recommend surgery in cases where the tumor is resectable and chemoradiation for locoregional control of the disease following surgery or when surgery is not feasible [2]. Studies have shown survival benefit in patients who undergo surgery and/or chemoradiation [3–7]. However, many patients with ATC already have advanced disease with distant metastases at the time of initial presentation, wherein the aforementioned treatment options may not be beneficial. Systemic treatment options for ATC patients with distant metastasis have been limited until the recent discovery of several kinase inhibitors (KI) with promising clinical benefit [8–11].

A better understanding of the molecular genomics of this tumor has led to the identification of several driver mutations in ATC [1, 12–14] such as *BRAF* and *RAS*. These are present in about 25–46% and 28% of tumors, respectively [1, 15, 16]. Recently, the FDA has approved the combination of dabrafenib and trametinib for the treatment of *BRAF* mutated ATC [17]. Lenvatinib is a multikinase inhibitor of VEGFR1–3, FGFR 1–4, PDGFR-α, RET and c-kit, approved by the FDA for the treatment of progressive radioiodine refractory differentiated thyroid cancer. Based on encouraging phase 2 results in Japan, the drug is now approved for ATC in that country [9]. In the Unites States, lenvatinib is currently being studied in clinical trials in the ATC population (NCT02657369).

Resistance to KI is a common problem in ATC and our understanding of mechanisms of resistance is limited [18]. There are limited treatment options for ATC patients whose disease progresses on KI. Immune deactivation of anti- tumoral responses has been suggested to play a role in solid tumors treated with KI [19, 20]. Several studies have attempted to characterize the type of immune cells and immune checkpoints present in the ATC tumor microenvironment particularly after treatment with multi-modal therapy and in the setting of kinase inhibitors [19, 21–23]. These studies have shown that ATC tumors express the PD-L1 on the tumor surface and that there is diffuse infiltration of the tumor with T-lymphocytes bearing PD-1 receptor [22]. Pembrolizumab is a monoclonal antibody against the PD-1 receptor approved by the FDA in the treatment of several cancers. Preliminary results from a phase 1 study with pembrolizumab in advanced differentiated thyroid cancers which progressed on standard therapies have shown promising results in term of clinical responses and overall survival [24]. In ATC, despite a low tumor mutation burden, a study reported partial responses in 2 out of 4 ATC patients treated with pembrolizumab [25]. However, in a clinical trial comprising of 30 ATC patients treated with single agent spartalizumab (anti-PD1), partial responses were observed in fewer than 20% of patients [26]. These responses are on the order of to those seen with systemic cytotoxic chemotherapy such as doxorubicin, paclitaxel and gemcitabine where partial responses were observed in 10–20% of cases [27]. Additionally, in our experience, patients progress rapidly when the KI is withdrawn. In our case report published recently [28], our patient progressed rapidly when he was taken off dabrafenib and trametinib at the time of post-operative radiation during which only single agent pembrolizumab was continued. However, on reintroducing the KI therapy, his tumor regressed again.

We sought to study the efficacy of adding pembrolizumab as a salvage therapy at progression in order to overcome resistance to KI in ATC.

## Methods

### Study population

Under an Institutional Review Board approved protocol, we queried our ATC database for patients who were initiated on treatment with combination pembrolizumab plus KI at the time of progression while on KI between August 2016 and August 2017 and who were followed at The University of Texas MD Anderson Cancer Center. All pathologic diagnoses of ATC were confirmed by a dedicated head and neck pathologist and all radiological images were reviewed by a single radiologist.

### Evaluations and definitions

Molecular testing on the tumor was done as a standard of care using either immunohistochemistry (IHC) and/or next generation sequencing (NGS) at our center (50 gene somatic mutation analysis panel or by Solid Tumor Genomics Assay v1 looking at 134 genes) or by Foundation One. PD-L1 status was determined by immunohistochemistry (IHC) on tumor tissue (clone 22C3, Dako) obtained at initial diagnosis, before initiation of KI. The efficacy of adding pembrolizumab to KI was determined by best overall response (BOR), progression free survival (PFS) and overall survival (OS). RECIST v1.1 was used to evaluate BOR. Clinical benefit was defined as stable disease (SD) plus partial response (PR). PET images, if available, were also reviewed for assessing metabolic response from baseline. PFS was defined as the time elapsed between adding pembrolizumab while on KI and progression or death whichever occurred first. OS was defined as the time elapsed between addition of the pembrolizumab to KI and death. Similarly, median OS was also calculated from the start of KI therapy until death. A single radiologist reviewed all cross-sectional images obtained at baseline and during treatment with pembrolizumab plus KI. Adverse events (AEs) were evaluated using Common Terminology Criteria for Adverse Events version 4.0 (CTCAE v.4.0) [29].

### Statistical analysis

The BOR for individual patients was calculated as percent change in the target lesions from baseline and depicted graphically as a waterfall plot. Kaplan Meier curves were used to describe median OS and PFS. Descriptive statistics were used to summarize patient characteristics and AEs. Statistical analyses were performed using SPSS version 22.

## Results

### Study population

Twelve ATC patients were treated with combination pembrolizumab plus KI at the time of clinical or radiological progression while on KI therapy, and were followed at our institution. The baseline characteristics are summarized in Table 1. Median age at addition of pembrolizumab was 60 years (range 47–84 years). Eight out of 12 (67%) patients were men. At the time of ATC diagnosis 3/12 (25%) patients were stage IVB and 9/12 (75%) stage IVC. All of them had either locoregional progression or appearance of new distant metastatic lesions on radiological staging scans while on KI. The site of metastases at addition of pembrolizumab included the lungs in all patients with stage IVC disease. Of these, one also had a cardiac metastasis which grew in size on KI. At the time of initiating pembrolizumab, 6 patients (50%) had an ECOG of 1, and 4 patients (33%) had an ECOG of 2. All patients’ tumor tissues were tested for the presence of *BRAF* V600E, either by IHC or NGS-50 gene somatic mutation analysis panel, 6 (50%) of which harbored a *BRAF* V600E mutation.

**Table 1:** Baseline characteristics of anaplastict thyroid cancer (ATC) patients treated with pembrolizumab added as a salvage therapy to kinase inhibitor therapy

	*N* = 12
Median Age at treatment start, years (range)	60 (47–84)
Gender, n (%)	
Men Women	8 (67) 4 (33)
Ethnicity, n (%)	
Caucasian Asian Black	9 (75) 2 (16) 1 (9)
Stage at Diagnosis, n (%)	
IVA IVB IVC	0 (0) 3 (25) 9 (75)
Pathology, n (%)	
ATC only Papillary thyroid cancer + ATC Poorly differentiated thyroid cancer + ATC Follicular thyroid cancer +ATC	4 (33) 4 (33) 3 (25) 1 (9)
PD-L1 status, n (%) < 10% 11–50% 51–100%	10 (83) 2 (20) 4 (40) 4 (40)
Performance Status, n (%)	
ECOG 0 ECOG 1 ECOG 2	2 (16) 6 (50) 4 (33)
Previous Treatment for ATC, n (%)^a^	
Surgery, n (%) Radiation/chemosensitizing, n (%) Bridging Chemotherapy, n (%)^b^	5 (41) 6 (50) 3 (25)
Targeted therapy^c^, n (%)	
Lenvatinib Dabrafenib + trametinib Trametinib alone	5 (41) 6 (50) 1 (9)

^a^Patients received more than 1 modality of therapy

^b^Chemotherapy as a bridging therapy while awaiting targeted therapy

^c^All patients with BRAFV600E mutations were treated with dabrafenib + trametinib

Prior treatment for ATC included surgery in 5 patients (44%), external beam radiation (EBRT) with or without radiosensitizing chemotherapy in 6 patients (50%), and 3 patients (25%) bridging chemotherapy with nab-paclitaxel with or without carboplatin, while awaiting mutation testing and procurement of the KI therapy. Details of the individual patients’ tumor genomics as well as the treatment modalities received have been described in Additional file 1: Table S1 and Table 2, respectively. PD-L1 status on the tumor, either primary or metastatic, was tested in 9 patients (75%) and have been described in Table 1.

**Table 2:** Patient characteristics: table below summarizes the individual patient characteristics

Patient	Age at start of P (y)	Stage at diagn-osis	Prior treatment before KI	PD-L1 status	KI	Time on KI alone (m)	Time on KI + P (m)	Change in target lesions and BOR on KI + P	PFS On KI + P (m)	OS from KI (m)	OS from KI+P (m)	Alive (A)/Deceased (D)
1	56	IVC	BC, CXRT	5%	DT	4.3	3.1	32% PD	0.7	7.4	3.1	D
2	68	IVC	BC	30%	DT	9.6	3.8	21% PD	1.5	13.4	3.8	D
3	57	IVB	Sg, CXRT	80%	L	1.9	6.9	19% PD	1.5	8.9	6.9	D
4	58	IVC	Sg, CXRT	5%	DT	26.2	13.9	−7% SD	0.6	40.1	13.9	A
5	60	IVC	BC	50%	L	5.5	15.8	−8% SD	12.8	21.4	15.8	A
6	47	IVB	Sg, CXRT	60%	DT	2.4	3	−14% SD	3	5.4	3	D
7	69	IVC	CXRT	n/A	DT	2	4.1	−19% SD	3.1	6.2	4.1	D
8	60	IVC	SC, BC	> 95%	DT	1.2	6.2	−35% PR	6.2	7.4	6.2	A
9	84	IVC	Sg-Lobectomy	> 10%	L	2.1	8.3	−45% PR	8.3	10.4	8.3	D
10	57	IVC	SC, XRT	n/A	L	2.4	16.1	−47% PR	13.1	18.5	16.1	A
11	73	IVC	Sg, RAI	20%	T	3.7	4.9	−48% PR	2.6	6.7	4.9	A
12	76	IVB	Sg, RAI	> 90%	L	1.5	5	−69% PR	5	5.8	5	D

*Sg* Surgery, *CXRT* Chemoradiation, *BC* Bridging chemotherapy with paclitaxel with or without carboplatin while awaiting KI, *SC* Systemic cytotoxic chemotherapy, *XRT* external beam radiation, *RAI* radioactive iodine, *DT* dabrafenib+trametinib, *L* lenvatinib, *T* trametinib alone, *w* weeks, *m* months, *y* years, *KI* kinase inhibitor, *BOR* best overall response, *PFS* progression-free survival, *OS* overall survival, *PD* progressive disease, *SD* stable disease, *PR*, partial response

Six patients (50%) who harbored the *BRAF* mutation were treated with a combination of dabrafenib and trametinib, 5 patients (41%) with lenvatinib and 1 patient (9%) was treated with single-agent trametinib. Of the 6 patients treated with a combination of dabrafenib and trametinib, 5 were on dabrafenib 150 mg twice daily plus trametinib 2 mg daily (full doses). The dose of lenvatinib was 24 mg (full dose) in two patients, 20 mg in two patients and 14 mg in one patient. The patient treated with single agent trametinib was started on 2 mg daily.

Median time from the start of KI therapy to addition of pembrolizumab was 9.6 weeks (95% CI = 8.1, 11.1; range 3–105 weeks). In the patients treated with dabrafenib plus trametinib, pembrolizumab was added after a median of 9.6 weeks (95% CI = 0, 20.5; range 4.9–105 weeks) while in those on lenvatinib, pembrolizumab was added after a median of 8.7 weeks (95% CI = 6.6, 10.86; range 3–22 weeks), *p* value = 0.24. Pembrolizumab was administered intravenously at a dose of 200 mg every 3 weeks.

### Efficacy of combined pembrolizumab and KI therapy

**Fig. 1:**
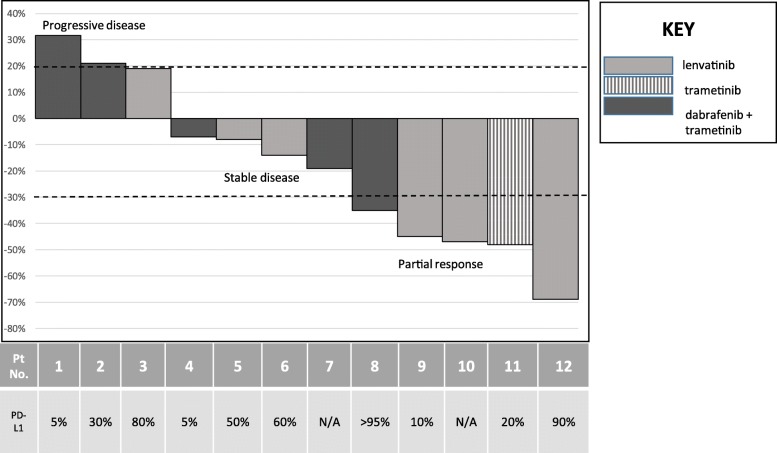
Waterfall plot demonstrating response to combination of kinase inhibitor therapy and pembrolizumab: A partial response was observed in 5/12 (42%), stable disease in 4/12 (33%) and progressive disease in 3/12 (25%). The box below the patient number describes the PD-L1 status on the tumor tissue

Median time on combined therapy was 5.6 months (range 2.9–15.8 months). All 12 patients on the combination of pembrolizumab and KI therapy were evaluable for BOR. In the entire cohort, 5/12 (42%) had PR, 4/12 (33%) SD and 3/12 (25%) PD. Overall, clinical benefit (PR + SD) was seen in 9/12 (75%) patients. The BOR is shown in the waterfall plot in Fig. 1. These responses were observed irrespective of their PD-L1 status on their tumor. Median time to BOR was 5.86 weeks (range 3–20 weeks). Partial response (PR) was the BOR in 1/6 (17%) patients on dabrafenib plus trametinib plus pembrolizumab (DTP), 3/5 (60%) patients on lenvatinib plus pembrolizumab and 1 patient on trametinib plus pembrolizumab. One patient who had a PR with a tumor regression of 47% on the combination of pembrolizumab and lenvatinib continues to have a response at the time of data analysis. His response to therapy has been shown in Additional file 2: Figure S1.

Stable disease (SD) was the BOR in—4/6 (67%) patients on dabrafenib, trametinib, pembrolizumab and 1/5 (20%) patient on lenvatinib plus pembrolizumab (LP). All patients with SD had tumor regression.

PD was the BOR in — 2/6 (33%) patients on DTP and 1/5(20%) patient on LP. Both had severe dysphagia from esophageal strictures requiring percutaneous gastrostomy tube placement. These patients were instructed to dilute the capsules in water and administer via gastrostomy tube. One patient was non-compliant with his oral KI which he found too cumbersome.

PETCTs were available in 11/12 patients for assessing response to combination pembrolizumab plus KI. Of these, complete metabolic response was seen in 2 patients at the time of last data analysis.

**Fig. 2:**
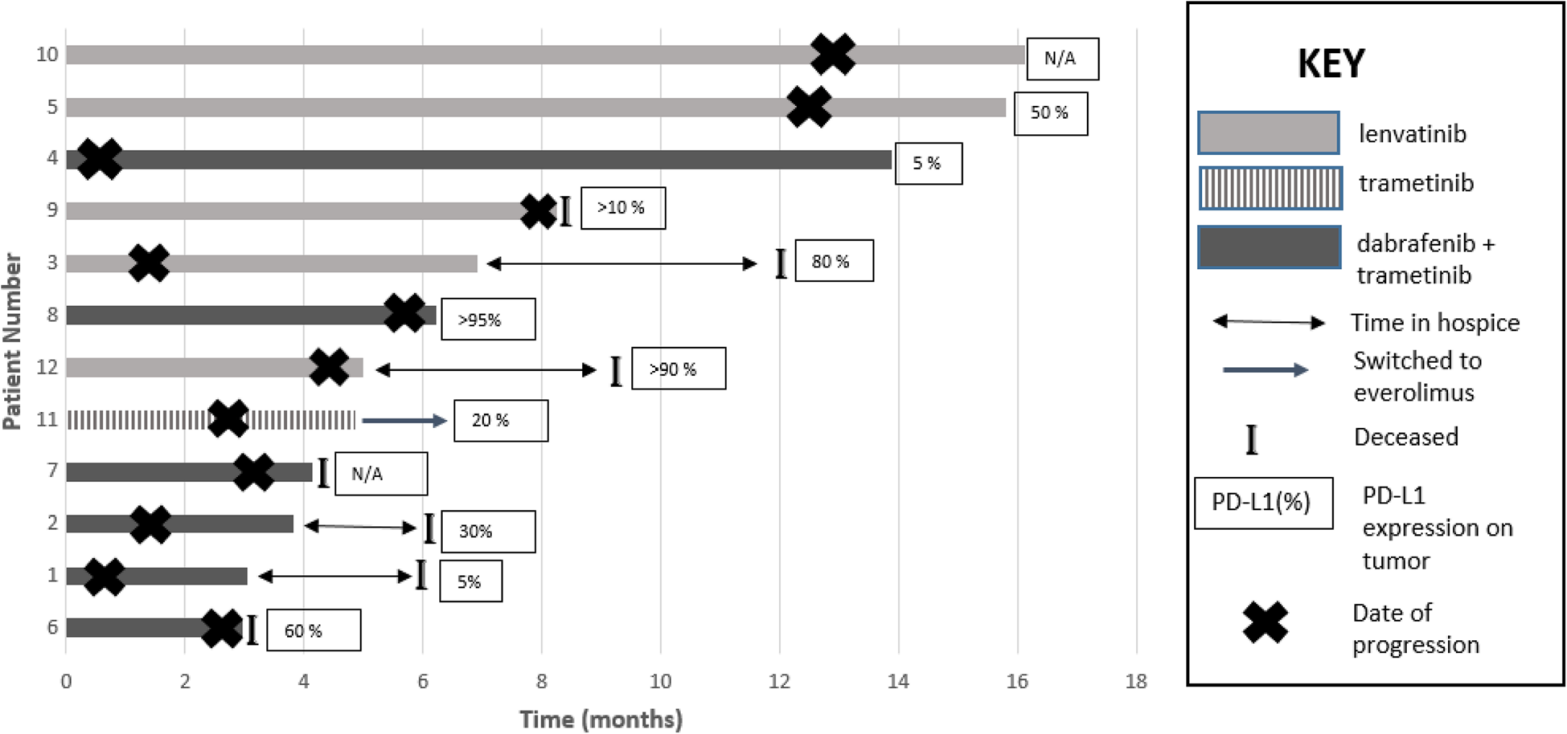
Swim lane plot demonstrating time on combination of kinase inhibitor therapy and pembrolizumab: The figure below describes the timeline on combination of kinase inhibitor therapy and pembrolizumab. Patient no. 11 was on trametinib with pembrolizumab. She developed Grade 3 rash on this combination and was switched to everolimus as a single agent. Seven patients (58%) died while on this therapy. Of these, four patients discontinued treatment due to adverse events and opted for hospice. The figure below shows that these patients continued to derive some survival benefit from exposure to this combination therapy and lived for a median of 4.25 months (range 2.29–5.4 months) after discontinuing all treatment for their cancer

The timeline of events for each patient from the initiation of combination pembrolizumab plus KI to the date of treatment discontinuation and/or last follow-up are described in Fig. 2.

Median PFS from the addition of pembrolizumab was 2.96 months (95% confidence interval (CI) = 2.2, 3.7, range 0.57–13.14 months) for the entire cohort. Patients were continued on the combination of KI and pembrolizumab at the time of progression.

### Survival analysis

**Fig. 3:**
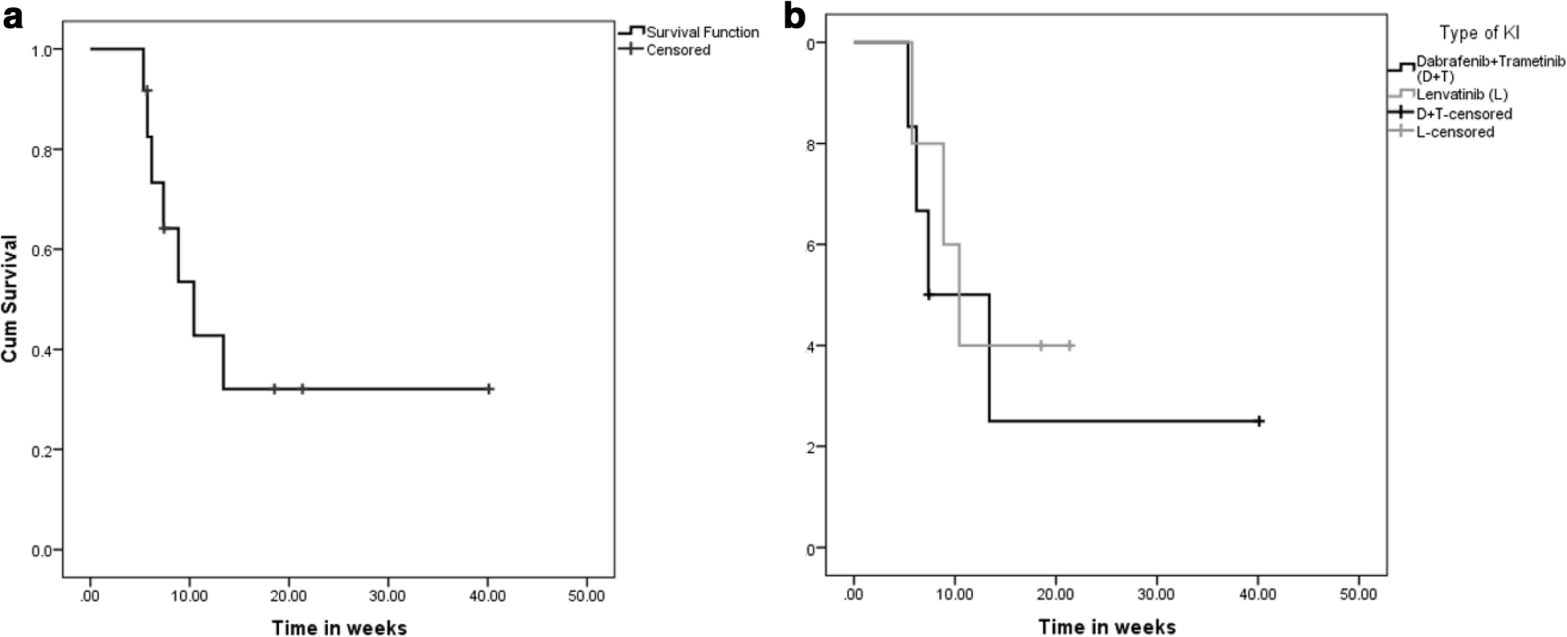
Overall survival (OS) from start of kinase inhibitor therapy. The figures below describe the median OS from the start of kinase inhibitor (KI) therapy. From the start of KI, the median OS was 10.43 months (95% CI = 6.02, 14.83) for the entire cohort (**a**). On the basis of type of KI (**b**), median OS was 7.4 months from the start of dabrafenib plus trametinib (95% CI = 0.43, 14.3), 10.4 months from the start of lenvatinib (95% CI = 7.1, 13.8). The patient on trametinib and pembrolizumab (Patient 11) was alive at the time of data analysis 6.7 months from the start of trametinib

**Fig. 4:**
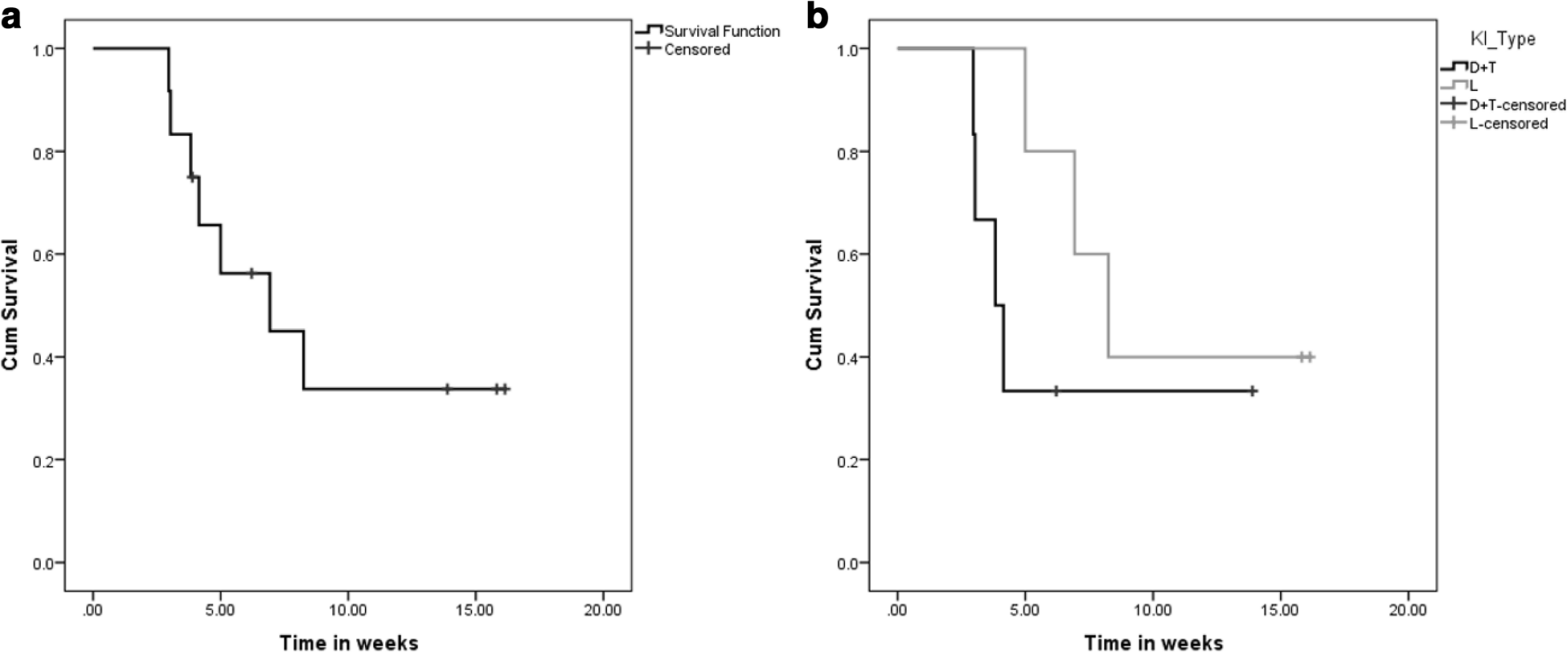
Overall survival (OS) after the addition of pembrolizumab. The figures below describe the median OS from the start of pembrolizumab added to their respective kinase inhibitor (KI) therapy. From the date of addition of pembrolizumab, the median OS was 6.93 months (95% CI = 1.7, 12.15) (**a**). In the patients who were on dabrafenib plus trametinib while on pembrolizumab, the median OS was 3.8 months (95% CI = 2.5, 5.1). Similarly the median OS of the patients who were on the combination of lenvatinib plus pembrolizumab was 8.25 months (95% CI = 5.4, 11.1), from the start of pembrolizumab (**b**). The patient on trametinib is alive 4.9 months after starting pembrolizumab in addition to her trametinib

From the start of KI, the median OS was 10.4 months (95% CI = 6.02, 14.83, range 5.4–40 months) for the entire cohort (Fig. 3a). On the basis of type of KI (Fig. 3b), median OS was 7.4 months from the start of dabrafenib plus trametinib (95% CI = 0.43, 14.3, range 5.4–40 months), 10.4 months from the start of lenvatinib (95% CI = 7.1, 13.8, range 5.8–21.4 months) and the patient who was started on trametinib was alive 6.7 months after its initiation. From the date of addition of pembrolizumab, the median OS was 6.93 months (95% CI = 1.7, 12.15, range 3–15.9 months) (Fig. 4a) in the entire cohort. The median OS was 3.8 months (95% CI = 2.5, 5.1, range 3–13.9 months) in dabrafenib plus trametinib plus pembrolizumab, 8.25 months (95% CI = 5.4, 11.1, range 5–16.14 months) in lenvatinib plus pembrolizumab and 4.9 months in trametinib plus pembrolizumab, respectively. (Fig. 4b). After a median follow up of 8.14 months (range 5.75–40.1 months) from the start of KI and 5.6 months (range 2.96–16.14 months) after the addition of pembrolizumab, 5 (42%) patients were alive.

Of the 7 patients who died, 4 patients had opted to discontinue all active treatment and opt for hospice and died a median of 3.3 months (range 2.3–4.2 months) after their last dose of pembrolizumab. Of the 3 patients who died while on combination of pembrolizumab and KI therapy, 1 patient on the combination of DTP died due to bleeding from a tracheo-innominate fistula. It was unclear if this was from tumor shrinkage versus PD. This patient had a history of prior surgeries, tracheostomy and external beam radiation to the neck. The other 2 died of PD.

Of the 5 patients who are alive at the time of data analysis, 2 of them had a complete metabolic response on PET-CT and 1 patient had complete metabolic response in distant metastases with persistent but stable residual avidity in the neck. These three patients have had an OS of over 12 months since the start of pembrolizumab.

### Safety and tolerability

AEs were as expected and manageable. All AEs reported have been described in Table 3.

**Table 3:** Adverse events (AEs). The table below lists the AEs on the combination of kinase inhibitor and pembrolizumab. The denominator used is the number of patients on the combination of lenvatinib (Len) with pembrolizumab, dabrafenib plus trametinib (D + T) with pembrolizumab and the patient on trametinib (tram) with pembrolizumab

Adverse event	All grades, n (%)			Grade 1, n (%)			Grade 2, n (%)			Grade 3, n (%)		
	Len *n* = 5	D + T *n* = 6	tram *n* = 1	Len *n* = 5	D + T *n* = 6	tram *n* = 1	Len *n* = 5	D + T *n* = 6	tram *n* = 1	Len *n* = 5	D + T *n* = 6	tram *n* = 1
Fatigue	5 (100)	5 (83)	1 (100)	1 (20)	3 (50)	1 (100)	3 (60)	1 (17)	1 (100)	1 (20)	0 (0)	0 (0)
Anemia	5 (100)	4 (67)	1 (100)	5 (100)	2 (33)	0 (0)	0 (0)	2 (33)	1(100)	0 (0)	0 (0)	0 (0)
Hypertension	5 (100)	2 (33)	1 (100)	3 (60)	2 (33)	1 (100)	2 (40)	0 (0)	0 (0)	0 (0)	0 (0)	0 (0)
Dry Mouth	5 (100)	2 (33)	1 (100)	5 (100)	2 (33)	1 (100)	0 (0)	0 (0)	0 (0)	0 (0)	0 (0)	0 (0)
Anorexia	5 (100)	1 (17)	1 (100)	3 (60)	1 (17)	1 (100)	2 (40)	0 (0)	0 (0)	0 (0)	0 (0)	0 (0)
Hoarseness	5 (100)	2 (33)	0 (0)	5 (100)	2 (33)	0 (0)	0 (0)	0 (0)	0 (0)	0 (0)	0 (0)	0 (0)
Dehydration	5 (100)	1 (17)	0 (0)	3 (60)	1 (17)	0 (0)	2 (40)	0 (0)	0 (0)	0 (0)	0 (0)	0 (0)
Hand-foot skin reaction	4 (80)	3 (50)	0 (0)	4 (80)	3 (50)	0 (0)	0 (0)	0 (0)	0 (0)	0 (0)	0 (0)	0 (0)
Rash	4 (80)	2 (33)	1 (100)	3 (60)	2 (33)	0 (0)	1 (20)	0 (0)	0 (0)	0 (0)	0 (0)	1 (100)
Pain	4 (80)	2 (33)	1 (100)	3 (60)	1 (17)	1 (100)	1 (20)	1 (17)	0 (0)	0 (0)	0 (0)	0 (0)
Hypokalemia	4 (80)	2 (33)	0 (0)	2 (40)	1 (17)	0 (0)	1 (20)	1 (17)	0 (0)	1 (20)	0 (0)	0 (0)
Weight loss	4 (80)	1 (17)	0 (0)	2 (40)	0 (0)	0 (0)	2 (40)	1 (17)	0 (0)	0 (0)	0 (0)	0 (0)
Diarrhea	4 (80)	1 (17)	0 (0)	3 (60)	1 (17)	0 (0)	1 (20)	0 (0)	0 (0)	0 (0)	0 (0)	0 (0)
Hypothyroidism	4 (80)	1 (17)	0 (0)	4 (80)	1 (17)	0 (0)	0 (0)	0 (0)	0 (0)	0 (0)	0 (0)	0 (0)
Mucositis	3 (60)	2 (33)	1 (100)	2 (40)	2 (33)	1 (100)	1 (20)	0 (0)	1 (100)	0 (0)	0 (0)	0 (0)
Dysphagia	3 (60)	3 (50)	0 (0)	2 (40)	1 (17)	1 (100)	1 (20)	2 (33)	0 (0)	0 (0)	0 (0)	0 (0)
Hyponatremia	3 (60)	2 (33)	0 (0)	3 (60)	1 (17)	0 (0)	0 (0)	0 (0)	0 (0)	0 (0)	1 (17)	0 (0)
Hyperuricemia	3 (60)	1 (17)	0 (0)	3 (60)	1 (17)	0 (0)	0 (0)	0 (0)	0 (0)	0 (0)	0 (0)	0 (0)
Hypomagnesemia	3 (60)	0 (0)	0 (0)	3 (60)	0 (0)	0 (0)	0 (0)	0 (0)	0 (0)	0 (0)	0 (0)	0 (0)
Fever	2 (40)	3 (50)	0 (0)	1 (20)	1 (17)	0 (0)	1 (20)	1 (17)	0 (0)	0 (0)	1 (17)	0 (0)
Nausea	2 (40)	2 (33)	1 (100)	1 (20)	2 (33)	1 (100)	1 (20)	0 (0)	0 (0)	0 (0)	0 (0)	0 (0)
Hair loss	2 (40)	1 (17)	1 (100)	2 (40)	1 (17)	1 (100)	0 (0)	0 (0)	0 (0)	0 (0)	0 (0)	0 (0)
Weakness	2 (40)	0 (0)	1 (100)	1 (20)	0 (0)	1 (100)	1 (20)	0 (0)	0 (0)	1 (0)	0 (0)	0 (0)
Hyperglycemia	2 (40)	2 (33)	0 (0)	2 (40)	1 (17)	0 (0)	0 (0)	1 (17)	0 (0)	0 (0)	0 (0)	0 (0)
Bleeding	2 (40)	1 (20)	0 (0)	2 (40)	0 (0)	0 (0)	0 (0)	0 (0)	0 (0)	0 (0)	0 (0)	0 (0)
Hyperbilirubinemia	2 (40)	1 (20)	0 (0)	1 (20)	0 (0)	0 (0)	1 (20)	0 (0)	0 (0)	0 (0)	0 (0)	0 (0)
Elevated alkaline phosphatase	2 (40)	1 (20)	0 (0)	1 (20)	0 (0)	0 (0)	1 (20)	0 (0)	0 (0)	0 (0)	0 (0)	0 (0)
Shortness of breath	1 (20)	0 (0)	1 (100)	1 (20)	0 (0)	1 (100)	0 (0)	0 (0)	0 (0)	0 (0)	0 (0)	0 (0)
Vomiting	1 (20)	1 (17)	0 (00	0 (0)	0 (0)	0 (0)	1 (20)	1 (17)	0 (0)	0 (0)	0 (0)	0 (0)
Transaminitis	1 (20)	0 (0)	1 (100)	0 (0)	0 (0)	1 (100)	1 (20)	0 (0)	0 (0)	0 (0)	0 (0)	0 (0)
Altered mental status	1 (20)	0 (0)	0 (0)	0 (0)	0 (0)	0 (0)	0 (0)	0 (0)	0 (0)	1 (20)	0 (0)	0 (0)
Lymphopenia	1 (20)	0 (0)	0 (0)	1 (20)	0 (0)	0 (0)	0 (0)	0 (0)	0 (0)	0 (0)	0 (0)	0 (0)
Hyperkalemia	1 (20)	0 (0)	0 (0)	1 (20)	0 (0)	0 (0)	0 (0)	0 (0)	0 (0)	0 (0)	0 (0)	0 (0)
Thrombocytopenia	1 (20)	0 (0)	0 (0)	1 (20)	0 (0)	0 (0)	0 (0)	0 (0)	0 (0)	0 (0)	0 (0)	0 (0)
Hypophosphatemia	1 (20)	0 (0)	0 (0)	0 (0)	0 (0)	0 (0)	0 (0)	0 (0)	0 (0)	1 (20)	0 (0)	0 (0)

Immune-mediated AEs were seen in 2 patients on combination of lenvatinib and pembrolizumab. Grade 2 colitis was seen in one patient treated with combination of pembrolizumab and lenvatinib and this was treated with budesonide without the need to hold pembrolizumab. Another patient developed grade 2 hepatitis after the second dose of pembrolizumab which required discontinuation of pembrolizumab and treatment with high dose prednisone.

## Discussion

We describe a series of 12 ATC patients who were treated with combination pembrolizumab plus KI as a salvage therapy added at the time of progression on KI therapy.

KI therapies targeting BRAF and MEK as well as those inhibiting VEGFR have shown promise in the management of ATC in the setting of a clinical trial as well as in the real world [8, 9, 11, 30]. While the median OS of ATC patients has improved on these therapies, these tumors eventually develop resistance resulting in progression of disease and death [11, 31]. Hence, there is a need to seek salvage therapies in these patients who progress on KI therapy, or use better combinational strategies upfront. In our study, the median OS was 6.9 months from the addition of pembrolizumab, as a salvage, to KI therapy at the time of progression. Due to the lack of a control arm, wherein patients who progressed on KI were not treated with the addition of pembrolizumab, we are unable to compare and definitively state what would be the effect of not starting pembrolizumab on OS at the time of progression. We did observe that patients who had a near complete or complete metabolic response on PET scans at restaging tended to have a longer OS compared to those who did not. This could suggest that the addition of pembrolizumab reduces the metabolic activity of the tumor. However, our numbers are too small to conclusively state a correlation between metabolic response and OS. This would be best explored in prospective clinical trials. To date, KI, especially BRAFi remains the best treatment option in metastatic ATC.

Several human ATC tissue analyses attempting to characterize the type of immune cells and immune checkpoints present in ATC microenvironment have been performed. These studies showed high PDL1 expression and high frequency of TILs [23]. Similar findings were reported in an ATC mouse model [19]. These data point to a hot immunogenic environment that can be targeted with immunotherapy. However, clinical data using immunotherapy in ATC patients is limited [32]. The combination of immunotherapy and KI has shown promise in the treatment of melanoma [33]. What is being explored currently is whether the combination of immunotherapy and targeted therapy is better tolerated and more efficacious when used simultaneously or sequentially [33]. While the use of anti-PD1 therapies such as pembrolizumab as a single agent needs to be explored in ATC, preclinical studies looking at the combination of KI and anti-PD1 therapy in ATC have suggested a benefit of using them together [19]. Single agent immunotherapy may not be useful as it takes time to start showing its effect as observed in advanced differentiated thyroid cancer tumors [24]. ATC is a rapidly growing tumor which can progress while awaiting the immunotherapy to take effect.

Research in melanoma has suggested the strategy of introducing anti-PD1 therapy before resistance to BRAF inhibitor therapy is expected to develop [33]. However, this needs to be prospectively explored in ATC. The majority of our patients derived clinical benefit from the combination of pembrolizumab added to their kinase inhibitor therapy at the time of progression on the latter. Although this strategy may provide additional benefit in some patients, prospective studies are needed to explore the timing of incorporating immunotherapy in the treatment of ATC patients. Mechanistic studies have suggested the addition of anti-PD1 therapy around the same time as initiation of KI therapy. In a murine model of ATC, the synergistic effect of combining anti-PD1 immunotherapy with BRAF inhibitor therapy has been shown to produce a significant tumor regression as the tumor cells bearing the *BRAF* V600E mutation tend to bear a higher expression of PD-L1 [19]. Additionally, the use of a BRAF inhibitor without the use of anti-PD1 inhibitor therapy showed increased expression of PD-L1 on BRAF wild type cells which interacts with the PD-1 on infiltrating T-cells inhibiting the anti-tumor immune response contributing to immune resistance and progression of disease. Similar changes in the tumor immune microenvironment have been shown in melanoma patients after 2 weeks of BRAF inhibitor therapy prior to clinical progression [34]. Therefore, by 9 weeks on dabrafenib and trametinib therapy the tumor microenvironment would be very different from what it was at the time of initiating KI therapy, suggesting maximal benefit obtained if pembrolizumab is added to KI earlier in the course of treatment. In a case report of an ATC patient treated with vemurafenib-(a selective BRAF inhibitor) and nivolumab (an anti-PD1 immunotherapy agent), the patient continued to have a response 20 months after starting nivolumab, which was started within days of starting vemurafenib at the earliest sign of progression [32]. Similarly, VEGF inhibition leads to hypoxia mediated increased expression of PD-L1 in certain tumors and has been proposed to have synergistic benefit when combined with anti-PD1 therapy [35, 36]. The combination lenvatinib and pembrolizumab has been shown to provide benefit in several solid tumors such as renal cell carcinoma (RCC) and non-small cell carcinoma [37]. We propose that ATC patients may benefit from addition of pembrolizumab to KI earlier in their course of treatment.

A study of 16 ATC patients treated with multimodal treatment reported a lower OS in patients with a PD-L1 expression of > 33% on their tumor [21]. In our case series, responses to anti-PD1 immunotherapy were seen irrespective of the PD-L1 expression on the tumor.

In terms of tolerability, the combination of kinase inhibitor therapy was associated with some grade 2 and grade 3 AEs which were immune-mediated and managed using standard treatment protocols outlined in CTCAE v 4.0. Drug discontinuation was required in 2 patients due to severe AEs such as grade 3 rash in the patient on trametinib plus pembrolizumab and grade 2 hepatitis in a patient on lenvatinib and pembrolizumab. Hepatitis is a commonly encountered AE when anti-PD1 therapy is combined with VEGFR inhibitors as seen in the clinical trials exploring this combination in RCC [37, 38]. Therefore, while responses with the combination of pembrolizumab and KI therapy are impressive, they are also associated with side effects which patients need to be educated about and for which they require close monitoring.

Limited by the retrospective nature of our study and a small sample size, prospective studies are needed to evaluate the correlation between PD-L1 expression, clinical response and survival in ATC. Considering this was a chart review based study, it is possible that several AEs were either under-reported or not graded uniformly and were subjective on the physician evaluating the AEs.

## Conclusion

Pembrolizumab could be used as a safe and effective salvage therapy to be added to kinase inhibitor therapy at the time of progression. Patients might benefit from the addition of pembrolizumab at the earliest sign of progression or earlier in the course of KI therapy in order to obtain maximum clinical and survival benefit from this combination therapy as the immune microenvironment may be less permissive at the time of progression on KI therapy. This combination should be prospectively explored in clinical trials. A trial exploring combination immunotherapy and targeted therapy is currently open and enrolling (NCT03181100).

## Additional files

Additional file 1: **Table S1.** This table describes the presence of mutations on the tumor tissue as well as in liquid biopsy of the patients in the series as well as the treatment received and their vital status (DOCX 28 kb)

Additional file 2: **Figure S1.** Patient number 10 who had a PR with a tumor regression of 47% on the combination of pembrolizumab and lenvatinib continues to have a response at the time of data analysis. Axial post contrast CT images through the chest demonstrates right lower lobe pulmonary nodule (red arrow) and pleural based left lower lobe nodularity (yellow arrow) on baseline scan(A) with treatment response on subsequent follow up imaging at 5 months (B) and 8 months (C). (PDF 256 kb)
