# Supplementary figures and images for: Salvage pembrolizumab added to kinase inhibitor therapy for the treatment of anaplastic thyroid carcinoma
Source: J Immunother Cancer. 2018 Jul 11;6:68. doi: 10.1186/s40425-018-0378-y (PMC6042271; doi:10.1186/s40425-018-0378-y)

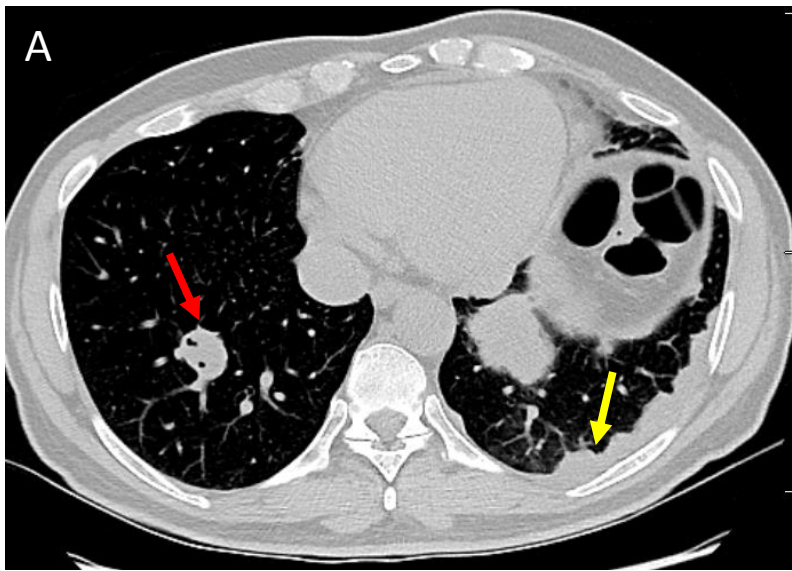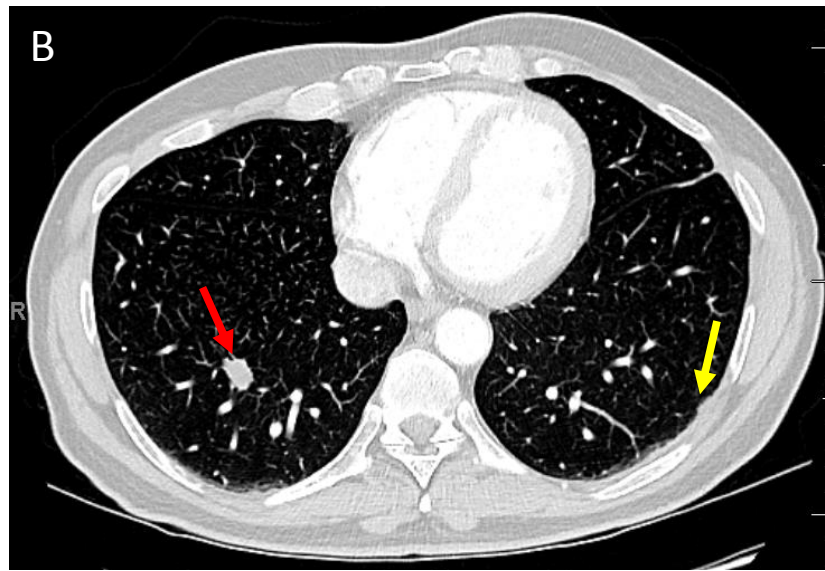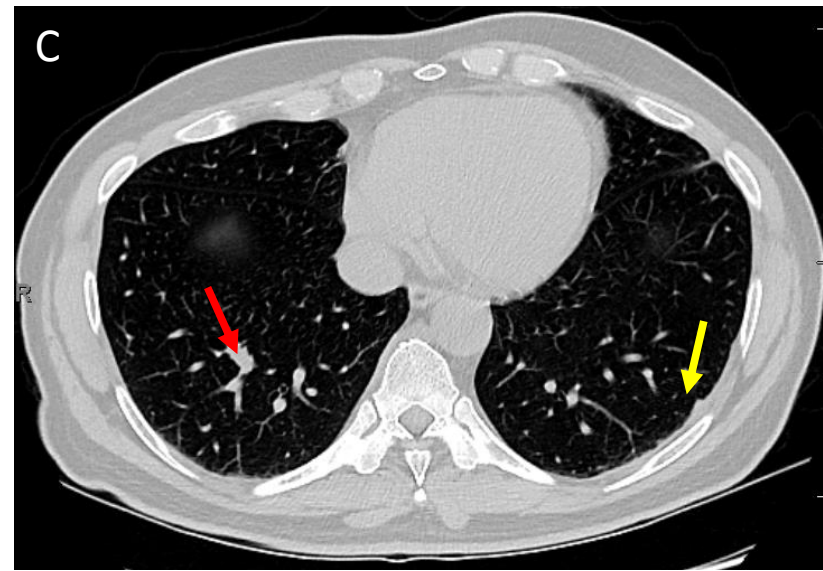

Supplement: Supplementary file 2 — Figure S1. Patient number 10 who had a PR with a tumor regression of 47% on the combination of pembrolizumab and lenvatinib continues to have a response at the time of data analysis. Axial post contrast CT images through the chest demonstrates right lower lobe pulmonary nodule (red arrow) and pleural based left lower lobe nodularity (yellow arrow) on baseline scan(A) with treatment response on subsequent follow up imaging at 5 months (B) and 8 months (C). (PDF 256 kb) [file 40425_2018_378_MOESM2_ESM.pdf]
