# Supplementary material for: Salvage pembrolizumab added to kinase inhibitor therapy for the treatment of anaplastic thyroid carcinoma
Source: J Immunother Cancer. 2018 Jul 11;6:68. doi: 10.1186/s40425-018-0378-y (PMC6042271; doi:10.1186/s40425-018-0378-y)
Supplement: Supplementary file 2 [file 40425_2018_378_MOESM1_ESM.docx]

													**Legend**					
**Patient Number**	**1**	**2**	**3**	**4**	**5**	**6**	**7**	**8**	**9**	**10**	**11**	**12**						
Age at KI start													41-50yrs	51-60yrs	61-70yrs	71-80yrs	81-90yrs	
Pathology													Pure ATC	ATC+PTC	ATC+FTC	ATC+PDTC		
Kinase inhibitor													lenvatinib	D+T	trametinib			
Response to therapy													PR	SD	PD			
Vital Status													Alive	Deceased				
PD-L1 expression													0-20%	21%-40%	41%-60%	61-80%	81-100%	Not checked
**Mutations on Tumor:**													**Mutations on Tumor:**					
BRAFV600E													Present	Absent				
p53													Present	Absent				
NRAS													Present	Absent				
PIK3CA													Present	Absent				
NF-1																		
PTEN													Present	Absent				
CDKN2A													Present	Absent				
HRAS													Present	Absent				
TERT													Present	Absent				
ATM													Present	Absent				
mTOR													Present	Absent				
CBL													Present	Absent				
PDGFRA													Present	Absent				
KIT													Present	Absent				
KDR													Present	Absent				
BRAFG469A																		
STK-11													Present	Absent				
**Mutations in cfDNA:**													**Mutations in cfDNA:**					
BRAF													Present	Absent	Not checked			
p53													Present	Absent	Not checked			
NRAS													Present	Absent	Not checked			
PIK3CA													Present	Absent	Not checked			
PDGFRA													Present	Absent	Not checked			
BRCA1													Present	Absent	Not checked			
NF-1													Present	Absent	Not checked			
PTEN													Present	Absent	Not checked			
KRAS													Present	Absent	Not checked			
RET													Present	Absent	Not checked			
TERT													Present	Absent	Not checked			
CCND2													Present	Absent	Not checked			
CDKN2A													Present	Absent	Not checked			
ATM													Present	Absent	Not checked			
GNAS													Present	Absent	Not checked			
KIT													Present	Absent	Not checked			
EGFR													Present	Absent	Not checked			
CTNNB1													Present	Absent	Not checked			
FGFR1 amplification													Present	Absent	Not checked			
RB1													Present	Absent	Not checked			
APC													Present	Absent	Not checked			
CDK6													Present	Absent	Not checked			
NTRK3													Present	Absent	Not checked			
AR													Present	Absent	Not checked			
